# Protocol for minimizing off-target neuronal activation during optical stimulation *in vivo*

**DOI:** 10.1016/j.xpro.2025.104342

**Published:** 2026-01-19

**Authors:** Simon Weiler, Mateo Vélez-Fort, Luke O’Hara, Stephen C. Lenzi, Eleni Maria Amaniti, Troy W. Margrie

**Affiliations:** 1 Sainsbury Wellcome Centre for Neuronal Circuits and Behavior, University College London, 25 Howland Street, W1T 4JG London, UK; 2 Neurobiological Research Facility, Sainsbury Wellcome Centre, University College London, London, UK

**Keywords:** Neuroscience, Behavior, Systems biology

## Abstract

*In vivo* optogenetic stimulation is a powerful approach for dissecting neuronal circuit function, yet it can inadvertently lead to activation of endogenous retinal opsins, producing off-target responses that propagate to the cortex and therefore confound experimental interpretation. Here, we present a protocol for optical device implantation in the mouse brain that minimizes light leakage from the implant and skull. We also describe *in vivo* electrophysiological techniques to assay off-target neuronal activity and ensure its amelioration.

For complete details on the use and execution of this protocol, please refer to Weiler et al.^1^

## Highlights

• Procedures for implanting optical devices and acute or chronic recording probes
• Instruction for reducing light emission from optical implants and the dorsal skull
• Steps to assess and minimize stimulation light-induced off-target neuronal responses
• Post hoc validation of the location of optical fiber and recording probe

## Abstract (editor highlights)

Publisher’s note: Undertaking any experimental protocol requires adherence to local institutional guidelines for laboratory safety and ethics.

## Graphical abstract

**Figure:**
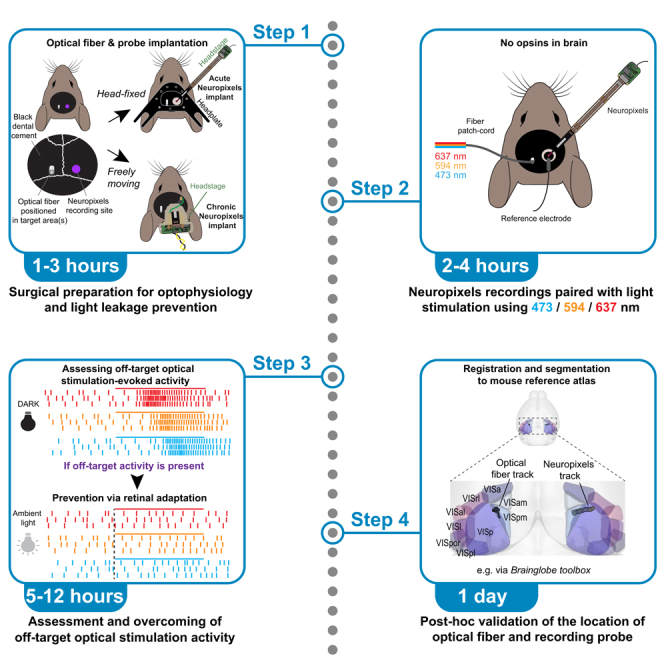

## Before you begin

**Figure 1:**
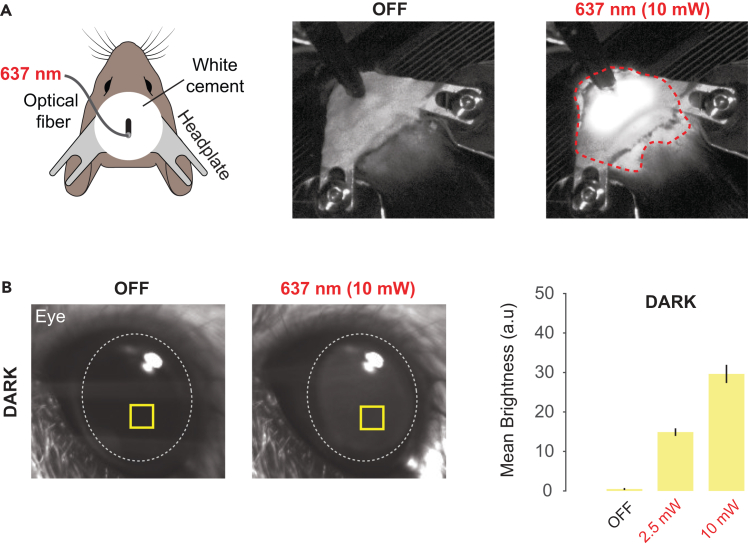
Primary sources of unintended off-target effects in intracranial optical stimulation (A) Direct light leakage from the implanted optical fiber, visible through the skull and cemented headplate. Here, red light is used as an example. Red dotted line indicates extent of light leakage. (B) Light propagation through brain tissue, detectable as it exits the eye using a camera. Here, red light is used as an example. The yellow square indicates the region where the baseline-subtracted mean pixel intensity (mean brightness) was measured across frames during light-off, 2.5 mW, and 10 mW conditions. The dotted line outlines the position of the animal’s pupil.

**Figure 2:**
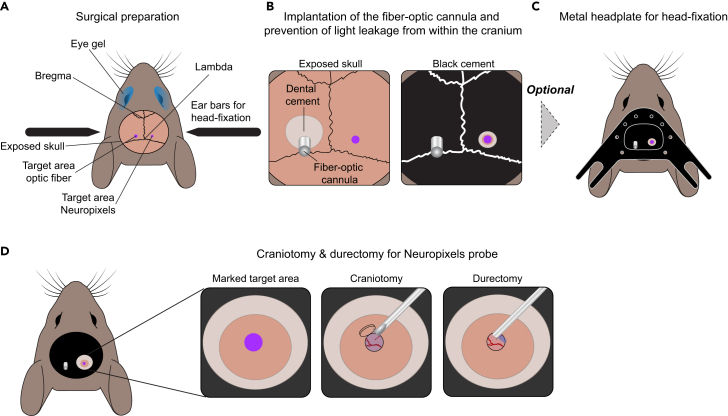
Major surgical steps for fiber-optic cannula implantation, preparation for Neuropixels probe installment and optional headplate mounting (A) Schematic illustrating stereotaxic coordinate marking. (B) Schematic displaying fiber-optic cannula implantation before and after application of black dental cement. (C) Schematic displaying a mounted headplate required for head fixation for acute recordings. (D) Schematic displaying craniotomy followed by durectomy in preparation for an electrophysiological probe recording.

Understanding the function of neuronal circuits *in vivo* often relies on optogenetic manipulation of neuronal populations using intracranial light stimulation at different wavelengths. However, recent experiments show that intracranial optical stimulation in mice can result in non-specific off-target activation of endogenous opsins located in the retina that potentially disrupts behavioral task performance.^2^ Such off-target effects can arise from two primary sources: 1) direct light leakage from the implant and exposed skull (Figure 1A) and 2) light propagation through brain tissue to the retina (Figure 1B).

Here we first describe a surgical approach for chronic implantation of an optical fiber positioned to optically stimulate a brain area of interest in mice lacking expression of any exogenous opsins. We detail the necessary steps needed to prevent light leakage from the craniotomy and exposed skull. We also provide detailed surgery approaches to perform acute and chronic extracellular electrophysiological recordings (Neuropixels 2.0^3^) and the experimental steps required for directly assessing potential light-induced off-target neuronal responses. Finally, we provide an example method for post hoc determination of the precise location of both the optical fiber and recording probe using the software tools *brainreg* and *brainglobe-segmentation*.^4^^,^^5^

### Prepare head-restriction and Neuropixels recording setup

**Timing: Up to 1 week**

1. Assemble rigid frame for head-restraining.
2. Place treadmill that allows the animal to be positioned comfortably during head-restrained acute experiments.
3. Assemble Faraday cage around the experimental apparatus to provide both electrical noise isolation and pitch darkness.
4. Place digital cameras and lenses for eye and body tracking (specification dependent on focal distance).
5. Prepare Neuropixels reference electrode (e.g., Ag/AgCl wire).
6. Assemble Neuropixels acquisition system (e.g., PXIe acquisition card inside a National Instruments chassis together with respective PCIe card in the computer).
7. Setup computer running the acquisition software (e.g., SpikeGLX or OpenEphys^6^).
8. Assemble 3- or 4-axis micro-manipulator.

### Calibration of laser power and ambient light source

**Timing: Up to 2 days**

9. Calibration of laser power.
  a. Connect the optical fiber cannula to the fiber patch-cord.
  b. Place the tip of the fiber in front of the digital power meter.
  c. Increase the voltage of the laser driver and take notes of the power meter readings.
  d. Repeat this process for each laser wavelength.
  e. Calculate the necessary voltage signal to the laser driver to obtain an input-output function (e.g., fiber tip powers of 1, 2.5, 5, 10 and 15 mW using a 200 μm diameter fiber). Calculate their respective irradiance using the online tool found at: http://web.stanford.edu/group/dlab/cgi-bin/graph/chart.php.
10. Calibration of ambient light source(s):
  a. Perform gamma correction when using monitor(s).
  b. For monitor(s) use the Psychotoolbox gamma correction tool.^7^ Follow the instructions prompted by the software.
  c. Use a luminance meter to determine the luminance values of the light source.
  d. At the end of the procedure, save the gamma correction table and use it when presenting isoluminant gray screens with Psychotoolbox (example code^8^).
  e. Determine the isoluminant gray values of your light source.
  f. With a lux meter, determine the brightness values required to obtain 20, 40 and 80 lux.

### Prepare the surgery area and surgical equipment

**Timing: 1–2 h**

11. Sterilization of surgical tools.
  a. Autoclave surgical tools: scissors, coarse and fine forceps, dental drill, cotton buds, 20 μl syringe tips, absorption spears and gown.
  b. Autoclave the aluminum head-plate tailored to the head-restraint setup.^9^
12. Gather materials supplied in sterile packaging: Histoacryl, resin dental cement Relyx Unicem 2, Dura-Gel, Silicon kwik-cast, 29G insulin syringes, surgical pen and scalpel.
13. Gather C&B Metabond and black dental cement.
14. Turn on the recovery rack (temperature at around 37°C).
15. For chronic Neuropixels recordings, setup micromanipulator and/or adapter for holding and inserting the probe within stereotaxic apparatus.

### Innovation

This protocol represents a significant innovation as it provides an *in vivo* electrophysiological method for detecting off-target neuronal activity caused by retinal activation of laser light propagating through brain tissue during optogenetic stimulation. Unlike previous approaches that measured off-target effects at the retina, this protocol directly focuses on off-target effects at the level of brain neuronal activity. This protocol also describes an easily implementable solution to overcome this off-target light-evoked neuronal activity.

### Institutional permissions

All experiments were performed in compliance with The Animal (Scientific Procedures) Act 1986, approved by the Sainsbury Wellcome Center’s Animal Welfare and Ethical Review Body (AWERB) and in accordance with ARRIVE guidelines.

Researchers utilizing this protocol must obtain approval from their institution’s Animal Ethics Committee.

## Key resources table

**Table:** 

REAGENT or RESOURCE	SOURCE	IDENTIFIER
**Deposited data**		

preprocessed data structure for Neuropixels recording	https://doi.org/10.6084/m9.figshare.27109249.v1	RRID:SCR_004328
data on light leakage	https://doi.org/10.6084/m9.figshare.30000826	SCR_004328
custom analysis code for Neuropixels recording	https://doi.org/10.5281/zenodo.13842774	SCR_002630
custom analysis code for light leakage	https://doi.org/10.5281/zenodo.17306097	SCR_002630

**Chemicals, peptides, and recombinant proteins**		

NaCl	Sigma-Aldrich	S9625
MgSO_4_	Sigma-Aldrich	M7506
CaCl_2_	Sigma-Aldrich	499609
Glucose	Sigma-Aldrich	G8270
KCl	Sigma-Aldrich	P9333
HEPES	Sigma-Aldrich	H3375
NaOH	Sigma-Aldrich	S8045
Agar	Sigma-Aldrich	A9793
DiI	Molecular Probes (Thermo Fisher Scientific)	V22885
Paraformaldehyde Solution, 4% in PBS	Thermo Fisher Scientific	15670799
Metacam	Boehringer Ingelheim	N/A
Isoflurane	Zoetis	N/A
Lubrithal	Dechra	N/A
Hibiscrub® 4% w/v cutaneous solution	Regent Medical Ltd.	N/A
Pentobarbital Sodium 10 mg/kg	Dolethal	N/A

**Experimental models: Organisms/strains**		

Mouse C57BL/6 (male and female, 7-36 weeks)	Charles Rivers Laboratories	N/A

**Software and algorithms**		

MATLAB 2024	https://www.mathworks.com/products/matlab.html	RRID:SCR_001622
Python (version 3.7.9)	https://www.python.org/	RRID:SCR_008394
SpikeGLX	https://billkarsh.github.io/SpikeGLX/	RRID:SCR_017041
OpenEphys	https://github.com/open-ephys/plugin-GUI	RRID:SCR_021624
Kilosort	https://github.com/cortex-lab/Kilosort	RRID:SCR_016422
Phy	https://github.com/cortex-lab/phy	N/A
Brainreg	https://github.com/brainglobe/brainreg	RRID:SCR_023858
brainglobe-segmentation	https://github.com/brainglobe/brainglobe-segmentation	RRID:SCR_023853
StitchIt	https://doi.org/10.5281/zenodo.3941901	N/A
BakingTray	https://github.com/SWC-Advanced-Microscopy/BakingTray	N/A
ScanImage v5.6	Vidrio Technologies, USA	RRID:SCR_014307
Laser irradiance calculation	http://web.stanford.edu/group/dlab/cgi-bin/graph/chart.php	N/A

**Other**		

637 nm laser source	Thorlabs	S4FC637
594 nm laser source	Coherent	OBIS 594 nm LS 60 mW
473 nm laser source	Coherent	OBIS 473 nm LX 150 mW
digital optical power meter	Thorlabs	PM100D
optical patch-cord	Newdoon	MM200/220 NA0.37
fiber-optic cannula	Newdoon	FOC-C-B-200-1.25-0.37-1.5
fiber-optic mating sleeve	Thorlabs	ADAL1
blackout tape (cover sleeve)	Thorlabs	T743-1.0
surgical scissors	Fine Science Tools	91500-09
coarse forceps	Fine Science Tools	11008-13
fine forceps 45 degree angled	Fine Science Tools	11251-35
disposable scalpels	Swann-Morton	7072312
surgical pen	Williams Medical	IDD5799/1
absorption spears	Sugi®	N/A
0.3 mm burr dental drill	Betts Metal Sales	Ball Tungsten Carbide Drill TOOLSTCR03
drill driver	Osada	LHP-12 160039
Isoflurane anesthesia system	Vet Tech	N/A
IVC cages	Tecniplast	GM500
stereotaxic apparatus	Benchmark	Benchmark
heated cage trolley	Vet-Tech	HE012
Stereoscope	Karl Kaps	SOM 82
body-heating mat apparatus	ALA Scientific Instruments	HEATINGPAD-1/2
Histoacryl	Braun	1050044
Clippers	Wella	Contura
dental adhesive resin cement	C&B	Super-Bond (Metabond)
black dental cement	Kemdent	Shade S123
Simplex Rapid Liquid	Kemdent	Simplex Rapid Liquid
LED UV curing light	Premium Plus	C01-M Mini LED Curing Light with 8mm Fibre Optic Light Guide
Catalyst V	Sun Medical Co.	Super-Bond
Relyx Unicem 2	3M Deutschland GmbH	56874
29G insulin syringes	BD Microfine	324891
Cameras	Basler AG	acA640-750um
2-lens camera objective	Custom from Thorlabs parts	AC127-025-B and AC127-075-B
Monitors	Magedok	MG300
luminance meter	Konica Minolta	LS-100
mouse treadmill	custom	N/A
head-restrain setup	custom	N/A
lux meter	Iso-tech	ILM-1337
Neuropixels	Imec	NP2014
PXIe and PCIe	National Instruments	PXIe and PCIe 8398
PXIe Chassis	National Instruments	PXIe-1071
Micromanipulators	Luigs & Neumann GmbH	N/A
NIDAQ	National Instruments	USB-6229
miniDAQ	National Instruments	USB-6002
Kwik-Cast	World Precision Instruments	N/A
grounding pin	World Precision Instruments	WPI 5482
grounding pin socket	World Precision Instruments	WPI 5483
silver wire	Advent Research Materials	Ag5489
Dura-Gel	Cambridge Neurotech	N/A

## Materials and equipment

**Table:** Cortex Buffer (1 L)

Reagent	Final concentration	Amount (g)
NaCl	125 mM	7.305
KCl	5 mM	0.3728
Glucose	10 mM	1.8016
HEPES	10 mM	2.383
CaCl_2_	2 mM	0.222
MgSO_4_	2 mM	0.2407

Add ∼500 μl of 1M NaOH as needed to adjust pH to 7.4 and an osmolarity of 290 mOsm.

***Note:*** Store Cortex Buffer at 4°C for up to a year.

• Prepare agar 3% using Cortex Buffer.

***Note:*** Prepare fresh on the day of acute recordings and discard after 24 h. Maintain agar 3% in its liquid form by keeping it at ∼70°C.

• Prepare Hibiscrub from 4% solution diluted 1:10 in autoclaved tap water to a final concentration of 0.4%.

***Note:*** Hibiscrub 0.4% should be stored at 18°C–20°C and discarded after 7 days.

• Prepare post-op analgesia jellies: Metacam (1.5 mg/ml) is diluted to 0.1mg/ml in Hartley’s strawberry flavour jelly.

***Alternatives:*** The reagents listed in key resources table have been tested successfully for the protocol described here. However, other commercial variants of these reagents exist and can also be used.

## Step-by-step method details

### Surgeries

**Timing: 3 h**

The aim of this step is to implant a fiber-optic cannula for delivery of laser light stimulus within the brain and perform a craniotomy/durectomy for either acute or chronic dense silicon probe electrophysiological recordings. Ensure that the surgery is performed in an aseptic environment to prevent post-op infections. Surgeries were performed on 7–36 weeks old C57BL/6 male and female mice.

**Figure 3:**
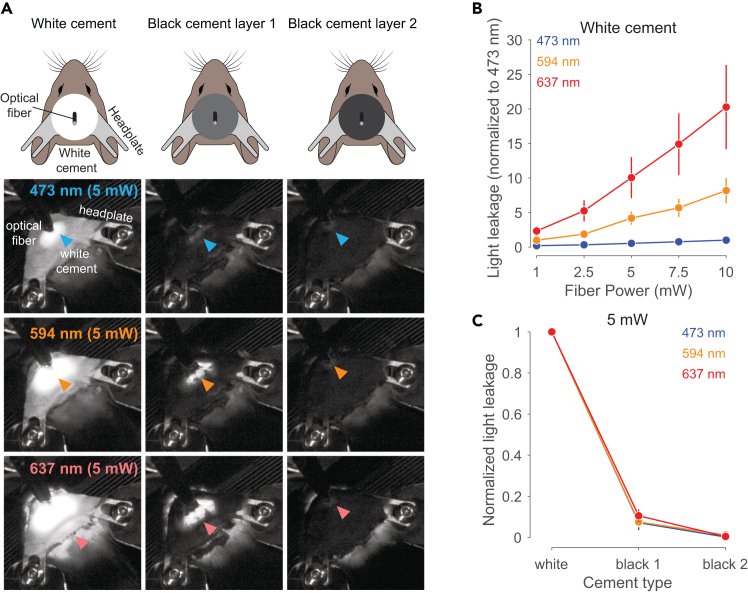
Application of black cement attenuates wavelength-dependent light leakage from the skull (A) First column: Light leakage through implant and skull with white dental cement for 473, 594 and 637 nm. Second column: Same as the first column with one layer of black cement added on top of the white cement. Third column: Same as the first two columns with two layers of black cement added. Arrowheads indicate area of light leakage. Note that light leakage was measured using an optical power meter placed above the head. (B) Average light leakage (mean ± sem) through the skull and implant for blue, orange and red laser stimulation across 1, 2.5, 5, 7.5 and 10 mW. Note that the light leakage is normalized to the leakage measured at 473 nm for the respective powers. (C) Normalized average light leakage (mean ± sem) through the skull after the application of white cement, or one or two layers of black cement, during blue, orange, and red laser stimulation at 5 mW.

**Figure 4:**
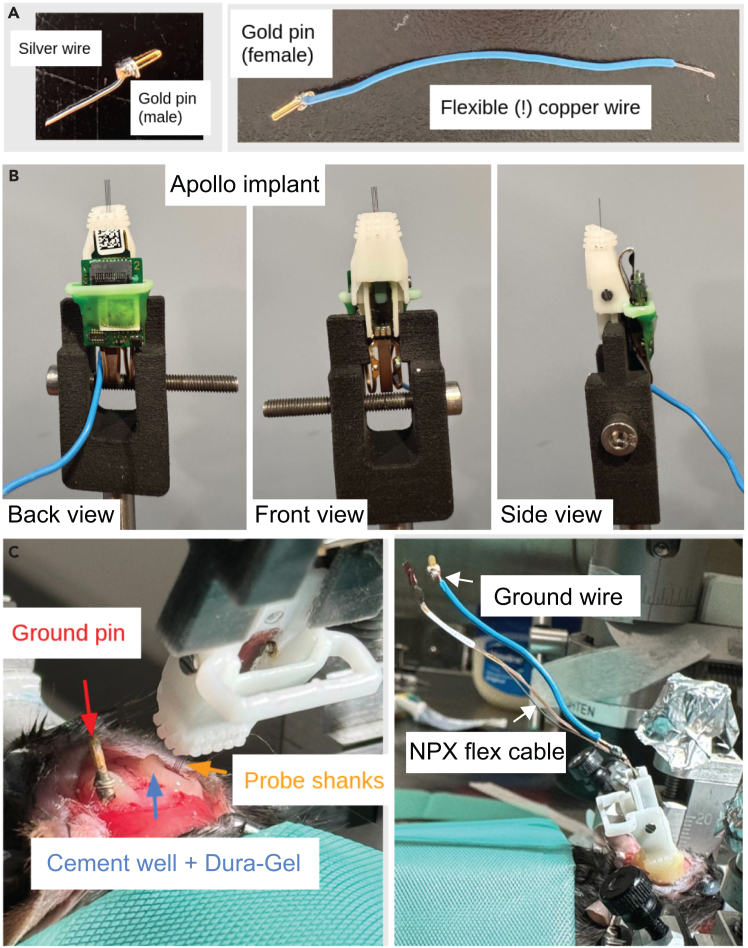
Key steps for chronic Neuropixels probe implantation (A) Preparation of a grounding pin and wires. (B) Assembled Apollo implant. (C) Implantation of Apollo implant for chronic Neuropixels recording. For purposes of improved visualisation, the implantation of an optical fiber and the application of black cement was not carried out.

**Figure 5:**
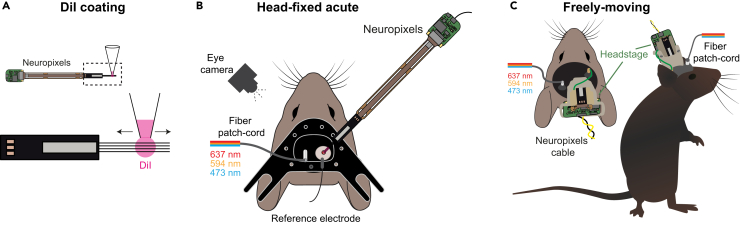
Acute head-fixed and chronic freely-moving Neuropixels recording (A) Schematic illustrating Neuropixels probe coating with DiI. Dashed box highlights zoom-in of DiI coating of probe shanks. (B) Schematic displaying the configuration for acute Neuropixels recordings paired with optogenetic stimulation in a head-fixed preparation. (C) Schematic displaying Neuropixels recordings paired with optogenetic stimulation in a freely-moving preparation.

See Figures 2, 3, 4, and 5 for a graphical demonstration of this stage.

1. Prepare mouse for surgical procedures.
  a. Insert fiber-optic cannula into fiber holder and place the holder on the stereotaxic frame.
  b. Initially anesthetize the mouse using isoflurane (4%–5%) inside an induction chamber (18x12x15 cm).
  c. Shave the dorsal surface of the head with clippers.
  d. Transfer the anaesthetized mouse to the stereotaxic frame (Figure 2A).
  e. Place anaesthetic mask over the snout and keep isoflurane level at ∼2% during rest of surgery.
  f. Apply eye gel Lubrithal (Figure 2A).
  g. Introduce the rectal probe and maintain body temperature at 37°C–38°C.
  h. Administer carprofen (5 mg/kg, s.c.).
    **CRITICAL:** During the entire surgery, regularly check the temperature of the animal, its respiratory rate (∼1 breath/s) and its eyes which should always be moist with eye gel. Follow institutional protocols and guidelines if respiratory rate or body temperature are not maintained.
  i. Disinfect the shaved area by gently using one-direction application of Hibiscrub 0.4% with a sterile cotton bud up to three times.
  j. Make a 1 cm antero-posterior incision through the midline of the head using a scalpel.
  k. Carefully remove the skin from the dorsal area where the optical fiber will be implanted using fine scissors.
  l. Scrape away all periosteal soft tissue overlying the parietal and interparietal bones.
  m. Dry the exposed bone with cotton buds and use the scalpel to roughen the bone surface.
  n. Zero the coordinates at bregma using the fiber holder on the stereotaxic frame. More specifically, first find the bregma position by lowering the fiber tip until it touches the bone and reset all coordinates. Then move to the lambda position, lower the fiber tip again, and ensure that the dorso-ventral (DV) value is 0 ± 10 μm.
  o. Mark the position of the brain area of interest, in this case the visual cortices on both hemispheres (e.g.,: antero-posterior (AP): −3.75 mm, medial-lateral (ML)= ±2.1 mm from bregma) using a surgical pen (Figures 2A and 2B).
2. Implant the fiber-optic cannula and prevent light leakage from the craniotomy and exposed skull.
  a. Drill a small circular (Ø = ∼1 mm) craniotomy on top of the marked area on one hemisphere.
  b. Once the bone is thinned, use forceps to lift and remove bone flap from the skull.
  c. Use a bent 29G syringe to gently tear and move the dura to the side of the craniotomy.
  d. Carefully lower the optical fiber into the brain at a speed of 10μm/s to the desired depth using a stereotaxic apparatus.
  e. Apply resin dental cement (Relyx Unicem 2) around the cannula and skull, then cure it with UV light of ∼500 mW/cm^2^ for 10 seconds to secure the cannula in place.
  f. Release the cannula from the fiber holder.
  g. Prepare C&B Metabond dental cement following the manufacturer instructions.
  h. Further fixate the cannula to the skull using C&B Metabond dental cement (Figure 2B).
  i. Prepare black dental cement following the manufacturer instructions.
  j. Apply two layers of black dental cement on top of the C&B Metabond dental cement without obscuring the area where the optical fiber will be connected to the optical patch-cord (Figure 2B).

**CRITICAL:** A minimum of two layers of black cement are necessary to prevent laser source light from exiting the head through the exposed skull. Aim for a ratio of 1:1:1 of dental cement, first layer and second layer of black cement. See Figure 3 and Araragi et al., 2021^10^ for a demonstration of the effectiveness of dental black dental cement in preventing light leakage.

***Note:*** Depending on the experimental condition either perform head-plate implantation for acute head-fixed experiments (step 3 and 4) or chronic Neuropixels implantation for freely moving experiments (step 5).

3. Implant the head-plate on the skull.
  a. Roughen the surface of the skull to provide a solid basis for cement to bind.
  b. Fix the metal head-plate to the skull using Histoacryl (Figure 2C).
  c. Prepare C&B Metabond dental cement following the manufacturer instructions.
  d. Use the dental cement to secure the head-plate in place.
  e. Again, apply two layers of black dental cement over the C&B Metabond dental cement sparing the craniotomy area (Figure 2C).
  f. Build a small well (3 mm in diameter, 2 mm in height) using C&B Metabond dental cement around the area where the craniotomy for acute recordings will be performed (Figure 2B).
    **CRITICAL:** Do not cover the area where the craniotomy for acute recordings will be performed.
  g. Cover the future craniotomy area with Kwik-Cast silicone sealant.
  h. Move the animal to its home cage and position the home cage on a preheated (∼37°C) recovery rack.
  i. Allow the animal to recover under supervision for at least 2 hours before removing the home cage from the recovery rack.
  j. Provide wet food and allow further recovery for at least 48 hrs.
    ***Note:*** Post-op analgesia can be provided with Metacam jellies. After letting it set overnight, 0.05ml/g body weight of the jelly is deposited in the animal’s home cage daily, for two days post-surgery.
    ***Note:*** The Kwik-Cast silicone can be kept on the animal for several weeks.
    ***Note:*** Animals can be grouped housed after surgery if needed.
4. Craniotomy/durectomy for acute recordings.
  a. After recovery of the animal, habituate the animal to the head-restriction setup with 2 to 3 sessions of head-restraint of 30 minutes each.
  b. On the day of acute probe recording, anesthetize the mouse using isoflurane 4%–5% in the induction chamber.
  c. Transfer the anaesthetized mouse to the stereotaxic frame.
  d. Place anaesthetic mask over the snout and keep isoflurane level at ∼2% during rest of surgery.
  e. Protect the eyes of the animal with eye gel Lubrithal.
  f. Introduce the rectal probe and maintain body temperature at 37°C–38°C.
  g. Administer carprofen (5 mg/kg, s.c.).
  h. Remove the Kwik-cast silicone sealant covering the bone.
  i. Drill a small circular (Ø = ∼1.5 mm) craniotomy using the dental drill on top of the marked area (Figure 2D).
  j. Once the bone is thinned, use forceps to lift and remove the bone flap from the craniotomy.
  k. Use a 29G syringe to gently tear and pull the dura to the side of the craniotomy (Figure 2D).
  l. Use a Pasteur pipette to perfuse the brain with the solution of Cortex Buffer.
    ***Note:*** At this stage, whiskers can be trimmed (typically to 2–5 mm long) to decrease whisker-related proprioception during the experiment.
    ***Note:*** For single shank recordings a small durectomy is still advised.
    **CRITICAL:** If there is bleeding, thoroughly wash with Cortex Buffer until the bleeding stops. Troubleshootingproblem 1.
  m. Immerse the surface of the brain with a thin layer of Cortex Buffer and seal the craniotomy with Kwik-cast silicone sealant.
  n. Return the animal to its home cage and on the preheated (∼37°C) post-op recovery rack.
  o. Allow the animal to recover for at least 2 hours under supervision before moving it to the recording setup.
    **CRITICAL:** Ensure that there is a thin film of Cortex buffer when applying the liquid silicone onto the brain surface.
5. Implantation of chronic extracellular probe.
  a. Prepare a grounding pin soldered to a short piece of silver wire cut such that it extends 1–2 mm beyond the base of the grounding pin (Figure 4A).
  b. Assemble the recording implant (e.g., the Apollo implant Figure 4B). Follow the instructions for printing and assembling Apollo implants (https://github.com/Coen-Lab/chronic-neuropixels). For further details on the usability of Apollo implants see Bimbard et al., 2024.^11^
  c. Drill a small (at least 0.5 mm) craniotomy away from the fiber and probe insertion locations and away from any blood vessels.
    ***Note:*** Typically, and where possible, drilling for all craniotomies should be done at the same time, i.e. before the probe, ground or fiber have been implanted to minimize the impact of pressure and vibrations on the skull once the fiber/ground and especially Neuropixels probe is in place.
  d. Either insert the silver wire approximately 1 mm into the brain or cement the ground pin inside a craniotomy onto the surface of the brain.
  e. Apply a small amount of resin dental cement (Relyx Unicem 2) around the ground wire and skull and then cure it with UV light to secure the ground wire in place before releasing (Figure 4C).
    ***Note:*** For optimal targeting it is important to ensure that bregma or a known reference coordinate is left accessible for later steps especially when aiming for deep structures.
    ***Note:*** Attach the headstage to the recording electrode and ensure it is functional before the surgery so everything can be implanted at once.
    **CRITICAL:** Roughen the surface of the skull to provide a solid basis for cement to bind.
  f. Drill a 1.5 mm craniotomy over the region of interest where the recording electrode will be targeted.
  g. Once the bone is thin, use a needle or forceps to gently lift and remove the bone flap from the craniotomy.
  h. Add a drop of Cortex Buffer to the craniotomy and then add a small amount of Kwik-Cast silicone sealant to the craniotomy to prevent it from drying out.
  i. Build a small well on the border of the craniotomy (∼0.5–1 mm in height) using dental cement (Figure 4C).
  j. Remove the Kwik-Cast silicone sealant.
  k. Use a bent 29G syringe to gently tear and place the dura to the side of the craniotomy (Figure 2D).
  l. Apply Dura-Gel to the craniotomy and wait for it to completely dry. The well should ensure that this does not spill over onto the skull.
    **CRITICAL:** Do not get Dura-Gel on the skull as this will prevent contact between cement and skull.
  m. Coat the recording probe with DiI (Figure 5A). For this, drag a drop of DiI (∼1μl) up and down the probe using a micropipette.
  n. Position the probe just above bregma. Zero the coordinates, move the probe to the craniotomy. See step 1n for detailed description on how to calibrate the stereotaxic coordinate system.
  o. Using a stereoscope, bring the tip of the probe flush to the pia.
  p. Insert the probe slowly (1–10μm/s^12^) into the region of interest using a motorized micromanipulator.
    **CRITICAL:** In the case of this type of Neuropixels probe it is important to ensure that all 4 shanks penetrate the tissue smoothly. Avoid blood vessels. Troubleshootingproblem 2.
  q. In the case of Neuropixels probe, apply resin dental cement (Relyx Unicem 2) to connect the skull and the external (docking) part of the Apollo implant and cure it immediately with the UV light.
  r. Make sure to cover the small protruding elements on the side of the implant.
  s. Repeat all around the implant until there are no gaps where the probe is exposed.
    ***Optional:*** For extra strength add a layer of C&B Metabond cement over the implant.
  t. Once the cement has dried, connect the grounding wire on the probe to the pin on the mouse.
  u. Cement the grounding pin and any exposed wire near the grounding pin.
    **CRITICAL:** Wait approximately 5 min for the cement to strengthen and take care not to apply shear forces to the pin or implant.
  v. Apply cement to cover the rest of the skull.
  w. Add two layers of black cement over the entire dorsal surface of the skull including around the electrophysiological implant.
  x. If there is excessive ground wire, tuck it gently around the implant and fix it in place with dental cement.

### Acute electrophysiology

**Timing: 2 h**

The following steps provide instructions on how to conduct an acute Neuropixels (in this case 4-shank) probe recording to assess off-target fiber-optic light stimulation of neuronal activity in the visual cortex. In this protocol we outline the assessment of off-target neuronal responses for acute experiments only (Figure 5B) but similar measurements can be obtained for chronic Neuropixels recordings (Figure 5C).

See Figure 5 for a graphical demonstration of this stage.

6. Penetration and stabilization of an acute recording probe.
  a. Head-restrain the habituated animal to the recording setup.
  b. Remove the Kwik-Cast silicone sealant using surgical forceps.
  c. Use a Pasteur pipette to perfuse the brain with Cortex Buffer.
  d. Fix the AgCl reference electrode into the recording well by securing the cable with removable adhesive putty.
  e. Connect the optical patch-cord.
    **CRITICAL:** To ensure a light tight connection between the fiber patch-cord and the optical fiber, shield the fiber-optic ferrule with blackout tape.
  f. Coat the electrophysiological probe with DiI (Figure 5A). For this, drag a drop of DiI (∼1μl) up and down the probe using a micropipette.
  g. Mount the probe on the micro-manipulators and connect to the headstage.
  h. Connect the AgCl reference electrode to the headstage.
  i. Using a stereoscope, bring the tip of the recording electrode flush to the pia.
  j. Lower the probe into the brain at a rate of 1–10 μm/s^12^ (Figure 5B).
  k. Start your recording software to visualize neuronal activity on the probe when penetrating the brain tissue.
    **CRITICAL:** Again if using this type of Neuropixels probe it is important to ensure that all 4 shanks penetrate the tissue smoothly. Avoid blood vessels. Troubleshootingproblem 2.
  l. Once the desired recording depth has been reached, carefully remove the Cortex Buffer with the tip of a tissue.
  m. Place a drop of liquified agar 3% onto the recording well, covering fully the brain surface and part of the AgCl reference electrode.
    ***Note:*** Ensure that the agar has cooled down (bearable to touch) before covering the brain surface with it.
  n. Once the agar solidifies, immerse with Cortex Buffer.
    **CRITICAL:** At this stage, the recorded electrophysiological signal-to-noise ratio should be of sufficient quality to resolve individual spikes.
  o. Wait 30 minutes for the brain tissue to settle after inserting the probe.
7. Setting up the camera and recording.
  a. Position the camera in front of either eye.
  b. Verify that the pupil is in focus.
  c. Start the recording software.
    **CRITICAL:** In order to synchronize the analog inputs/outputs, electrophysiological and camera recordings, ensure that a common digital input clock is recorded on all 3 main components of the setup.
  d. Check the laser source is ready to provide light stimulation by measuring the output intensity after the patch-cord and optical fiber using a digital power meter.
  e. Check that the laser can be driven by the analog or digital input.
  f. Check the camera is ready to record by triggering it with a digital input and observe whether frames are being captured.
  g. Begin the electrophysiological recording.
  h. Begin the optical stimulation protocol.
8. Assess off-target stimulation using extracellular electrophysiological recordings of neuronal activity.
  a. With the ambient light source (e.g., computer monitors) turned OFF, present a minimum of 5 laser pulses of your desired wavelength(s) in blocks of increasing laser intensities (e.g., 1, 2.5, 5, 10 and 15 mW).
  b. Repeat the step above with the ambient light source turned ON and for different isoluminant gray values (e.g., 20, 40 and 80 lux).
  c. Stop the electrophysiological recording.
  d. Retract the probe at ∼100 μm/s rate.
  e. Apply Kwik-Cast silicone sealant directly on top of the Cortex Buffer and Agar to seal the craniotomy.
  f. Release the animal’s head-restraint and place it in its home cage.

***Note:*** To achieve best recording quality, only one penetration per craniotomy is advised.

### Localization of probe and optical fiber tracks

**Timing: 1 day**

To determine the precise positioning of the recording electrode(s), the following steps should be followed. Note that the automated whole-brain imaging using serial two-photon tomography (STPT^13^^,^^14^) presented here can be replaced with clearing and light sheet microscopy or by cutting slices of the brain using a vibratome, then mounting brain sections on histology slides and locating the silicon probe track (DiI, red) in the brain using a simple fluorescent microscope.

**Figure 6:**
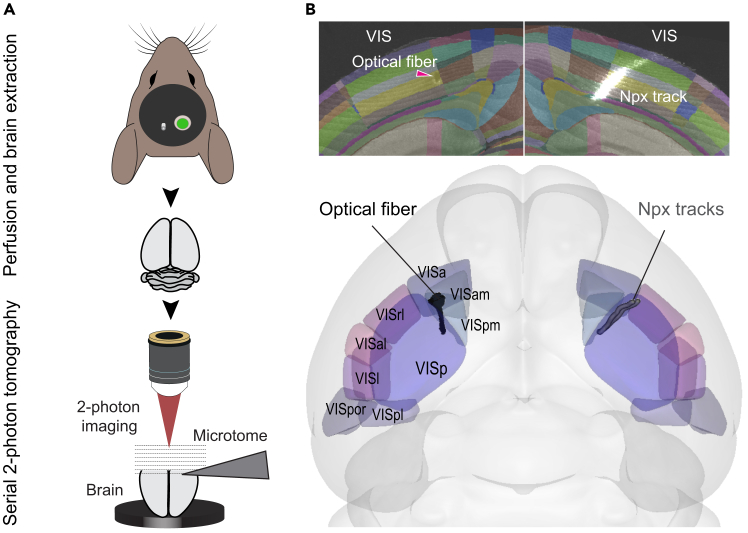
Three-dimensional rendering of implants registered to the mouse reference atlas (A) Schematic depicting brain extraction and subsequent automated serial 2-photon tomography used for whole-brain imaging. (B) Top: Whole brains are mapped onto the Allen Mouse Brain Common Coordinate Framework (CCFv3^15^) and the optical fiber track (magenta arrowhead) as well as the Neuropixels tracks are segmented. Bottom: Visualisation of segmented optical fiber and Neuropixels tracks warped to the common reference atlas coordinate space in 3D along with primary and higher visual cortical areas.

See Figure 6 for a graphical demonstration of this stage.

9. Whole-brain imaging.
  a. Perform transcardial perfusion of the animal using 10 ml of 100 mM PBS followed by 10 ml of saline containing 4% PFA. For a detailed perfusion protocol, see Wu et al, 2021^16^: https://bio-protocol.org/epdf/3988.
  b. Dissect brain out and submerge in fixative overnight at 4°C.
  c. After 24 hrs wash brain in PBS solution at least twice and store in PBS at 4°C.
  d. Mount the fixed brain on a STPT microscope (Figure 6A). For a protocol for STPT whole-brain imaging, please follow the steps outlined here: https://bakingtray.mouse.vision/users/user_guide.
10. Localization of probe and optical fiber.
  a. Register the images of the brain using *brainreg*.^4^^,^^5^
  b. Locate the DiI signal and the lesion left by the optical fiber on the registered brain (Figure 6B).
  c. Use the *brainglobe-segmentation*^5^ tool to reconstruct the probe and optical fiber track (Figure 6B).
  d. Use the output of *brainglobe-segmentation* to confirm the localization of the recording probe within the brain.

***Note:*** At this stage, electrophysiological parameters can also be used to verify the localization of the probe in the brain. See Tyson at al., 2022^5^ for details.

11. Analysis of data collected.
  a. Use Kilosort 2.0^17^ and Phy integrated in the pipeline by Velez-Fort et al, 2025^18^ for cluster analysis.

## Expected outcomes

**Figure 7:**
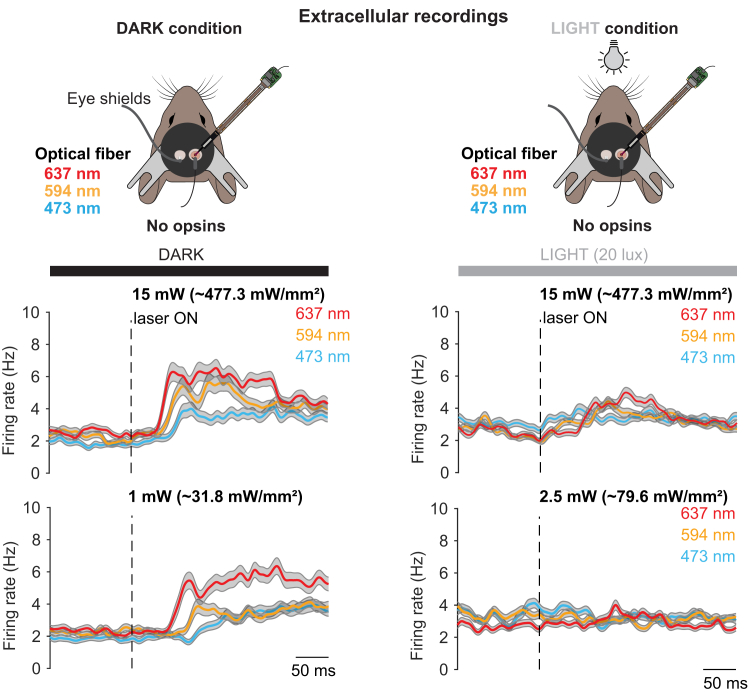
Assessment of off-target stimulation through extracellular probe recordings of neuronal activity Schematic of the recording setup. Red (673 nm), orange (594 nm) or blue (473 nm) laser is delivered via an optical fiber implanted in the left visual cortex. Translaminar Neuropixels recordings are performed in the contralateral visual cortex in complete darkness (left) and under ambient light (20 lux delivered via two monitors displaying a full screen iso-illuminant gray image, right). Average spike density functions for all units aligned to the onset (dotted line) of red, orange or blue laser stimulation (n = 761 cells, n = 4 mice) using either 15 mW (top) or 1 mW (bottom). Grey shaded area indicates sem. Schematics and traces adapted from Weiler et al. 2024.^1^

In a single session, this protocol allows the recording of several tens to hundreds of neurons across the cortical depth. The neuronal activity can then be aligned to the onset of laser light stimulation in dark conditions, or with ambient 20, 40 or 80 lux isoluminant gray presented on two monitors (Figure 7). In a typical recording, the activity of visual cortical neurons recorded in darkness is expected to increase for all wavelengths (473, 594 and 637 nm) and at all intensities we have tested (1, 2.5, 5, 10, 15 mW; Figure 7A). Given the fact that steps have been taken to minimize laser light leakage from the dorsal skull (Figures 1A and 3) and that videography of the eye reveals laser light escaping the brain through the pupil (Figure 1B), it is likely that intracranial laser light activates endogenous opsins present in the animal’s retina, which in turn activates the visual pathways.^1^^,^^2^

However, for experimental conditions where isoluminant gray screens (20 lux) were presented in front of the animal, the off-target retinal stimulation induced brain activity is expected to be fully abolished for blue (473 nm) and orange (594 nm) wavelengths (Figure 7). In the case of the red (637 nm) laser, off-target effects are expected to be abolished at lower laser intensities (1 or 2.5 mW). For 5 and 10 mW red laser intensities, only when 80 lux of ambient light was used did we observe abolishment of retina-evoked cortical activity. At 15 mW, 80 lux of ambient light failed to prevent off-target red light activation of the visual system.^1^

## Limitations

This protocol assesses off-target light stimulation effects on brain activity recorded in the visual cortex. It does not however provide any indication of the effect of intracranial optical stimulation on brain areas downstream from the main visual pathway(s). Since the off-target effects shown here in the visual cortex are strong and the main visual pathways are highly interconnected with other areas such as the retrosplenial cortex, auditory cotex and superior colliculus it is highly recommended that one should assess the impact of off-target effects on their brain area of interest - particularly in regions where visual integration is known to occur.

The protocol described here shows that gray screens at 20 lux (in the case of blue and orange lasers) provide enough luminance to leverage retinal light adaptation and abolish off-target laser stimulation effects on visual cortical activity. However, lower luminance bounds were not tested and it is possible that even lower ambient luminance levels are effective in abolishing off-target effects.

## Troubleshooting

### Problem 1

Bleeding during craniotomy/durectomy (related to step 4k and step 5k).

When removing the bone flap during the craniotomy or tearing the dura during the durectomy, blood vessels might be pierced. Small to medium bleeding can be stopped.

### Potential solution

The solution is to avoid piercing blood vessels: use the drill at a shallow angle and progressively thin the bone avoiding suddenly breaking through it. During the durectomy, use a 29G needle that has been bent against a hard (and sterile) surface to produce a “hook-like” tip. Pierce the dura at the edge of the craniotomy that has no visible vessels. If bleeding occurs despite the precautionary steps described above, use a Pasteur pipette to thoroughly wash the surface of the brain with Cortex Buffer. Absorb the excess of Cortex Buffer and blood with absorption spears and repeat the process until bleeding stops.

### Problem 2

Difficulty in getting the 4 shank Neuropixels probe to penetrate smoothly through brain tissue (related to step 5p and 6e).

The Neuropixels probe shanks have sharp tips and should penetrate the brain tissue smoothly. In some cases however the presence of dura that was not retracted during the durectomy or dry brain tissue may not allow one or several shanks to penetrate the tissue. In these cases, use a sharpened recording probe to insert through the dura.

### Potential solution

Use Cortex Buffer to keep the surface of the brain continually moist. If dry, the superficial layers of the brain will be damaged and it seems to also make smooth probe penetration more difficult. Descending and retracting the probe shanks against brain tissue several times can also help to puncture any dura that has been left over post-durectomy.

Finally, the shanks can be further sharpened by holding them at a shallow angle (∼30 degrees) against a fine rotating sander (the rotating platter of a discarded Hard Drive or a micro-grinder such as the Narishige EG-45 can be used) and then carefully washed. Sharpening the probe tips also minimizes tissue drag during penetration and therefore, can only ameliorate recording quality.

## Resource availability

### Lead contact

Further information and requests for resources should be directed to and will be fulfilled by the lead contact, Simon Weiler (s.weiler@ucl.ac.uk).

### Technical contact

Technical questions about performing this protocol should be directed to and will be answered by the technical contact, Simon Weiler (s.weiler@ucl.ac.uk).

### Materials availability

No new mouse lines or materials were generated for this protocol.

### Data and code availability

• The data for the experiments on light leakage through the implant and the eyes can be found at https://doi.org/10.6084/m9.figshare.30000826.
• The corresponding analysis code can be found at https://doi.org/10.5281/zenodo.17306097.

## Acknowledgments

The authors are grateful to the support staff of the Neurobiological Research Facility at Sainsbury Wellcome Center (SWC). This research was funded by the Sainsbury Wellcome Centre core grant from the Gatsby Charitable Foundation (GAT3361) and Wellcome Trust (219627/Z/19/Z), a Wellcome Trust Investigator Award (214333/Z/18/Z) and Discovery Award (306384/Z/23/Z) to T.W.M., and by SWC core funding to the Neurobiological Research Facility. S.W. was funded by a Feodor-Lynen fellowship from the Alexander von Humboldt Foundation.

## Author contributions

S.W., M.V.-F., and T.W.M. conceived the project. S.W. and M.V.-F. performed acute electrophysiology experiments. S.W. and L.O. performed surgeries and experiments to assess light leakage through the skull. S.C.L. performed chronic Neuropixels probe implantation. S.W. analyzed all data. S.W., M.V.-F., E.M.A., L.O., and T.W.M. wrote the manuscript.

## Declaration of interests

The authors declare no competing interests.
